# Inhibitors of the *Candida albicans* Major Facilitator Superfamily Transporter Mdr1p Responsible for Fluconazole Resistance

**DOI:** 10.1371/journal.pone.0126350

**Published:** 2015-05-07

**Authors:** Mikhail V. Keniya, Edmond Fleischer, Anette Klinger, Richard D. Cannon, Brian C. Monk

**Affiliations:** 1 The Sir John Walsh Research Institute, School of Dentistry, University of Otago, Dunedin, New Zealand; 2 MicroCombiChem e.K., Wiesbaden, Germany; Institute of Microbiology, SWITZERLAND

## Abstract

**Objective:**

To identify a novel class of inhibitors of fungal transporters involved in drug resistance.

**Methods:**

A series of structurally-related low molecular mass compounds was synthesized using combinatorial chemistry of a cyclobutene-dione (squarile) core. These compounds were screened for their inhibition of plasma membrane Major Facilitator Superfamily (MFS) and ATP-binding cassette (ABC) transporters responsible for efflux pump-mediated drug resistance in the fungal pathogen *Candida albicans*. Strains of *Saccharomyces cerevisiae* that specifically overexpress the MFS pump CaMdr1p or the ABC transporter CaCdr1p were used in primary screens and counterscreens, respectively, and to detect inhibition of glucose-dependent Nile Red efflux. Efflux pump inhibition, activity as pump substrates and antifungal activity against yeast and clinical isolates expressing efflux pumps were determined using agarose diffusion susceptibility assays and checkerboard liquid chemosensitization assays with fluconazole.

**Results:**

The screen identified five structurally-related compounds which inhibited CaMdr1p. Two compounds, A and B, specifically chemosensitized AD/CaMDR1 to FLC in a pH-dependent fashion and acted synergistically with FLC in checkerboard liquid MIC assays but compound B had limited solubility. Compound A chemosensitized to FLC the azole-resistant *C*. *albicans* strain FR2, which over-expresses CaMdr1p, inhibited Nile Red efflux mediated by CaMdr1p but not CaCdr1p and was not toxic to cultured human cells. A minor growth-inhibitory effect of B on AD/CaMDR1, but not on AD/CaCDR1 and AD/CaCDR2, indicated that compound B may be a substrate of these transporters. The related compound F was found to have antifungal activity against the three pump over-expressing strains used in the study.

**Conclusions:**

Compound A is a ‘first in class’ small molecule inhibitor of MFS efflux pump CaMdr1p.

## Introduction

The azole resistance of *Candida albicans* clinical isolates can be caused by several mechanisms. These include over-expression of, or mutations in, the drug target lanosterol 14α-demethylase, other changes in sterol metabolism and energy-dependent drug efflux [1,2]. There are two classes of efflux pump involved in *C*. *albicans* azole resistance: ATP-binding cassette (ABC) transporters, such as CaCdr1p, powered by ATP hydrolysis; and major facilitator superfamily (MFS) transporters, for example CaMdr1p, that utilise the plasma membrane electrochemical gradient to translocate substrates [2]. The *C*. *albicans* MFS transporter gene *MDR1* (also named *BEN* ^*r*^) was identified by its ability to confer benomyl and methotrexate resistance on *Saccharomyces cerevisiae*. Although azole-susceptible *C*. *albicans* clinical isolates usually show low-level constitutive expression of CaCdr1p [3], azole-resistant clinical isolates often overexpress one or more efflux pumps including CaCdr1p, CaCdr2p and CaMdr1p [4–9]. Inactivation of CaMDR1 was reported to markedly reduce virulence of *C*. *albicans* in an animal model [10] but subsequently *MRR1*-dependent activation of CaMDR1 expression was found not to increase pathogenicity [11]. High-level azole resistance most often correlates with overexpression of CaCdr1p, and to a lesser extent CaCdr2p, but in some isolates CaMdr1p is the only pump overexpressed [3,4,7]. This overexpression of CaMdr1p is usually due to mutations in the transcriptional regulator Mrr1p [12,13]. Overexpression of CaMdr1p confers intermediate-level resistance to the triazole drug fluconazole (FLC) [14,15] while overexpression of CaCdr1p is associated with efflux of a wider range of substrates. This difference in substrate specificity is not surprising considering the structural and mechanistic differences between ABC and MFS pumps.

It has been proposed that pump inhibitors could be used to chemosensitize resistant *C*. *albicans* strains to azoles, thus lowering the dose of antifungal required for therapy, potentially minimizing side-effects and making the selection of drug resistant strains less likely [2,16–18]. Several studies have investigated inhibitors of ABC efflux pump CaCdr1p [18–22]. There are very few reports, however, of inhibitors of CaMdr1p [23,24]. We previously used CaMdr1p as a counterscreen to identify RC21v3, a chemosensitizer specific for CaCdr1p [18]. In the present study we were interested in identifying inhibitors of CaMdr1p and we used a *S*. *cerevisiae* strain expressing CaCdr1p as a counterscreen to test the specificity of the CaMdr1p hits. These hits were also tested for their ability to inhibit CaCMdr1p-mediated Nile Red efflux [25] specifically and chemosensitize to FLC *C*. *albicans* clinical isolates that express single or multiple classes of efflux pump. Inhibitors of Mdr1p will be of value in studying pump function and may have therapeutic potential for infections caused by *C*. *albicans* strains expressing this transporter.

## Materials and Methods

### Strains and media

The *S*. *cerevisiae* host strain AD 1-8u^-^ (AD) used for pump overexpression (Table 1) is hypersusceptible to xenobiotics because 6 major plasma membrane transporters and one major vacuolar ABC transporter are deleted [26]. In addition, this host strain is deleted of the gene encoding the transcriptional regulator Pdr3p while the *pdr1-3* gain-of-function mutation results in constitutive high-level transcription from the *PDR5* promoter. Although the endogenous MFS transporter ScFlr1p (orthologue of CaMdr1p) is not deleted in AD, the 250-fold greater susceptibility of AD to FLC than the strain overexpressing CaMdr1p means that the endogenous ScFlr1p activity can be ignored for most purposes. Transformation cassettes containing the *CaCDR1*, *CaCDR2* and *CaMDR1* genes and the empty cassette with *URA3* marker (from pABC3) were used to transform *S*. *cerevisiae* AD by integration at the *PDR5* locus [26]. Synthetic defined medium (SD) which contained 0.74 g/L Complete Supplement Mixture (CSM; Formedia, Hunstanton, UK), 6.7 g/L Yeast Nitrogen Base without amino-acids (BD, Sparks, MD, USA) and 20 g/L dextrose, was prepared without pH adjustment (initial pH ~ 6.0). ‘SD pH 6.8’ medium was SD medium containing 10 mM MES and 20 mM HEPES buffered to pH 6.8 with Tris. The SD media were used for strain maintenance and growth susceptibility assays.

**Table 1:** Yeast strains used in this study.

Yeast	Strain	Description	Reference
***S*. *cerevisiae***	AD/pABC3	*URA3*-containing cassette control	[26]
	AD/CaMDR1A	strain overexpressing CaMdr1p	[26]
	AD/CaCDR1B	strain overexpressing CaCdr1p	[26]
	AD/CaCDR2A	strain overexpressing CaCdr2p	[26]
***C*. *albicans***	FHB1 (TL1)	FLC-susceptible clinical isolate	[27]
	FHB3 (TL3)	FLC-resistant derivative of FHB1 overexpressing CaCdr1p and CaCdr2p	[27]
	SGY-243	FLC-susceptible clinical isolate derivative (low constitutive CaCdr1p expression)	[28,29]
	FR2	FLC-resistant derivative of SGY-243 overexpressing CaMdr1p	[29]

The *C*. *albicans* strains used in this study are listed in Table 1. FHB1 (TL1) and FHB3 (TL3) (kindly provided by Prof. T.C.White) are isogenic clinical isolates from the same patient [27]. FHB3 daughter strain showed a CaCdr1p - CaCdr2p dependent azole drug resistance (MIC_FLC_ = 64μg mL^-1^ measured in accordance with CLSI) versus azole sensitive parent FHB1 (MIC_FLC_ = 0.5μg mL^-1^) [3]. FR2 was prepared from its isogenic clinical isolate derivative SGY-243 [28] *(ade2/ade2* Δ*ura3*::*ADE2*/Δ*ura3*::*ADE2*, (MIC_FLC_ = 0.5μg mL^-1^ *)* after culturing in medium containing FLC [29]. The resistance of FR2 to FLC (MIC_FLC_ = 32μg mL^-1^) has been shown to be primarily CaMdr1p-dependent [29]. FHB1 and FHB3 were cultured in SD media while SGY-243 and FR2 were grown in SD medium supplemented with uridine (80 mg/L).

### Compound preparation

A combinatorial library of compounds was prepared by MicroCombiChem e.K. (Wiesbaden, Germany) using click chemistry methods. All compounds were purified by high performance liquid chromatography to at least 95% purity and validated by mass spectrometry (HPLC-MS). Samples of 3–5 mg were dissolved in DMSO to 10 mM and stored at -20°C in aliquots until use. Samples were thawed at room temperature, and briefly heated to 40°C, if required, for compound dissolution.

### Disk diffusion assays

Yeast strains were grown to late-logarithmic phase in SD or SD pH 6.8 medium. A portion of the cell culture (0.4 mL) was mixed rapidly with 20 mL SD containing 0.6% agarose at 49°C and then poured on 20 mL of the same medium already solidified in a rectangular Petri dish (80 mm x 120 mm). The OD_600nm_ of the yeast cells in the overlay was 0.08 (equivalent to ~8 x 10^4^ CFU/mL, ~870 CFU/cm^2^). For chemosensitization assays, FLC (Claris Lifesciences, Ahmedabad, India) was added to the medium at 1/8 MIC for *S*. *cerevisiae* strains or 1/4 MIC for *C*. *albicans* strain*s*. Sterile 6 mm BBL paper disks (Becton, Dickinson and Company Sparks, MD, USA) preloaded with 5 μl of test compound were placed onto the solidified top agarose. A control agarose plate without FLC was used to detect antifungal activity of the compounds. Plates were incubated at 30°C for 48–72 h and photographed using a digital camera. The D-octapeptide derivative RC21v3 (2 nmol/disk) was used as a positive control for chemosensitization of cells expressing CaCdr1p [18]. The D-decapeptide inhibitor of the plasma membrane H^+^-ATPase BM2 (3 nmol/disk) provided a positive control for fungicidal activity [17]. The effect of compound A on membrane potential was tested by loading 200nmol of hygromycin B (Sigma, St Louis, MO, USA) on a paper disk in the centre of a seeded agarose plate. Disks containing compound A were positioned 15mm from the centre.

### Checkerboard liquid susceptibility assay

A late-logarithmic phase culture of the yeast strain overexpressing CaMdr1p was diluted in SD media pH 6.8 to OD_600nm_ = 0.02 (~3.3 x 10^5^ CFU/mL), distributed in a 96-well microtitre plate containing FLC (0–490 μM) and test compound (0–80 μM or to solubility limit). The plates were incubated with shaking at 30°C. Optical density readings at 600 nm were obtained after 48 h. To determine whether the drug combination had a fungistatic or fungicidal effect, samples (5 μL) taken from the wells with no visible growth were applied to SD agar plates either neat or after 40-fold dilution. The compound was considered to be fungicidal when less than 1% of the input cells gave CFUs.

The synergism of selected compounds with FLC was measured quantitatively using FIC (fractional inhibitory concentration) values. The FIC for FLC (FIC_F_) was calculated using the formula FIC_F_ = MIC_F/C_/MIC_F_ where MIC_F/C_ is the minimum growth inhibitory concentration (MIC) of FLC in the presence of the compound C and MIC_F_ is the MIC for FLC alone [30]. FIC_C_ (MIC_C/F_/MIC_C_), where MIC_C/F_ was the MIC of compound C in the presence of FLC, was also estimated. FIC values given as <x indicate that the MIC_C_ was not detected at the highest concentration of compound tested. The FIC index (FICI) was defined as FIC_F_ + FIC_C_. In accordance with published guidelines [31], an FICI of ≤ 0.5 was considered to indicate synergism. The FICI was given as a less than a maximum value (<x) for hits where MIC_C_ was not detected due to compound insolubility.

### Nile Red pumping assay

The efflux activity of *S*. *cerevisiae* cells overexpressing CaMdr1p or CaCdr1p was evaluated using a previously reported Nile Red pumping assay [25]. Each strain was grown in SD pH 6.8 to OD_600nm_ ~2–3. The cells were washed with sterile PBS, resuspended in PBS at OD_600nm_ ~2 and starved overnight on ice. The starved cells were washed, resuspended at OD_600mn_ = 1 in 5 mM 2-Deoxy-D-Glucose (DOG, Sigma, USA) buffered with HEPES-NaOH pH 7.0 and incubated at 30°C for 30 min with gentle agitation. The cells were loaded with Nile Red (Sigma, USA) by incubating them with the dye (7.5 μM) at 30°C for 30 min. The cells were washed twice with cold HEPES-NaOH pH 7.0, resuspended at OD_600nm_ = 10 and kept on ice for up to 2 h. Pumping assays were carried out in triplicate in 96-well microtitre plates. Samples of compound A (100 μL) were serially diluted to give final concentrations in the assay from 0.3 to 10 μM. Cell suspensions (50 μL) were added to each well and the plate positioned in a Synergy 2 plate reader (BioTeK, Winooski, VT, USA) to pre-warm at 26°C for 3 min. Pumping was initiated by adding 50 μL of 80 mM glucose, or 50 μL of 80 mM DOG as a control. Fluorescence measurements (filter set Ex 485/20, Em 528/29) commenced about 15 sec after glucose addition (T_0_) and then at 30 sec intervals for 11 minutes, with 5 sec of shaking between each measurement. As aqueous phase Nile Red is less fluorescent than the cell-bound dye, its efflux was detected as a decrease in fluorescence. Compound A has significant fluorescence at the wavelength used to measure Nile Red. The Nile Red fluorescence was therefore expressed as % of the fluorescence at T_0_.

### Toxicity towards human cells

Toxicity towards cultured human epithelial cells (HEp2, NCTC, Porton Down, UK) was measured as described previously [32]. In brief, cultured HEp2 cells were incubated for 4 or 24 h in the presence of drug, washed and stained using the LIVE/DEAD Viability/Cytotoxicity Assay Kit (Invitrogen, Life Technologies, Auckland, NZ). The images were captured using a Zeiss 510 LSM Axiovert 200M inverted confocal laser scanning microscope. Eight areas of 0.04 mm^2^ were analysed for live, metabolically active cells (visible with green fluorescein optical filter only); dead, permeable cells (visible with red rhodamine optical filter only) and; damaged cells (visible in both channels).

## Results

### Screening for inhibitors of CaMdr1p

**Fig 1:**
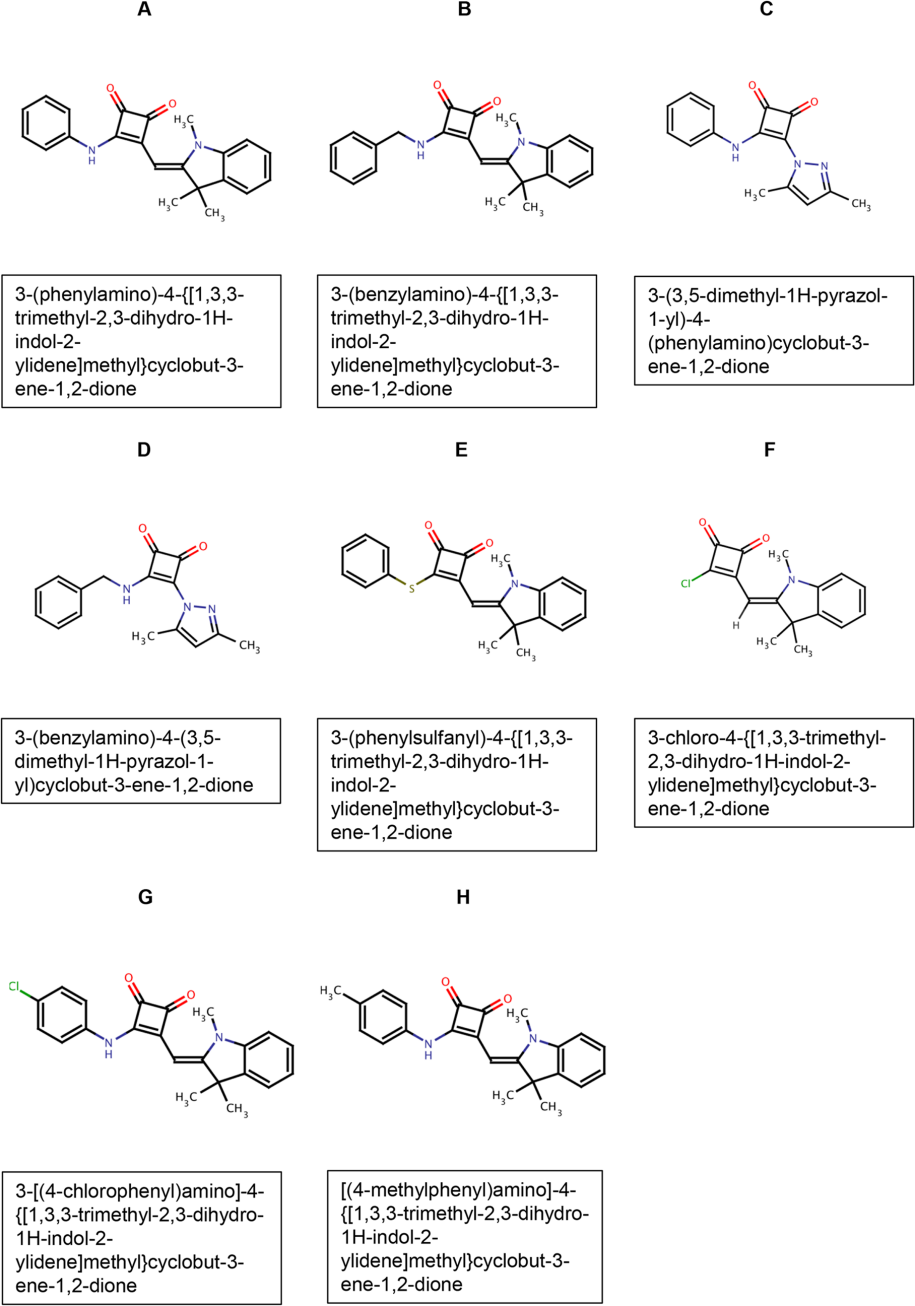
Compound structures. The compound name (A-H, see Table 2) is shown above each structure and its full IUPAC name in the box underneath.

**Fig 2:**
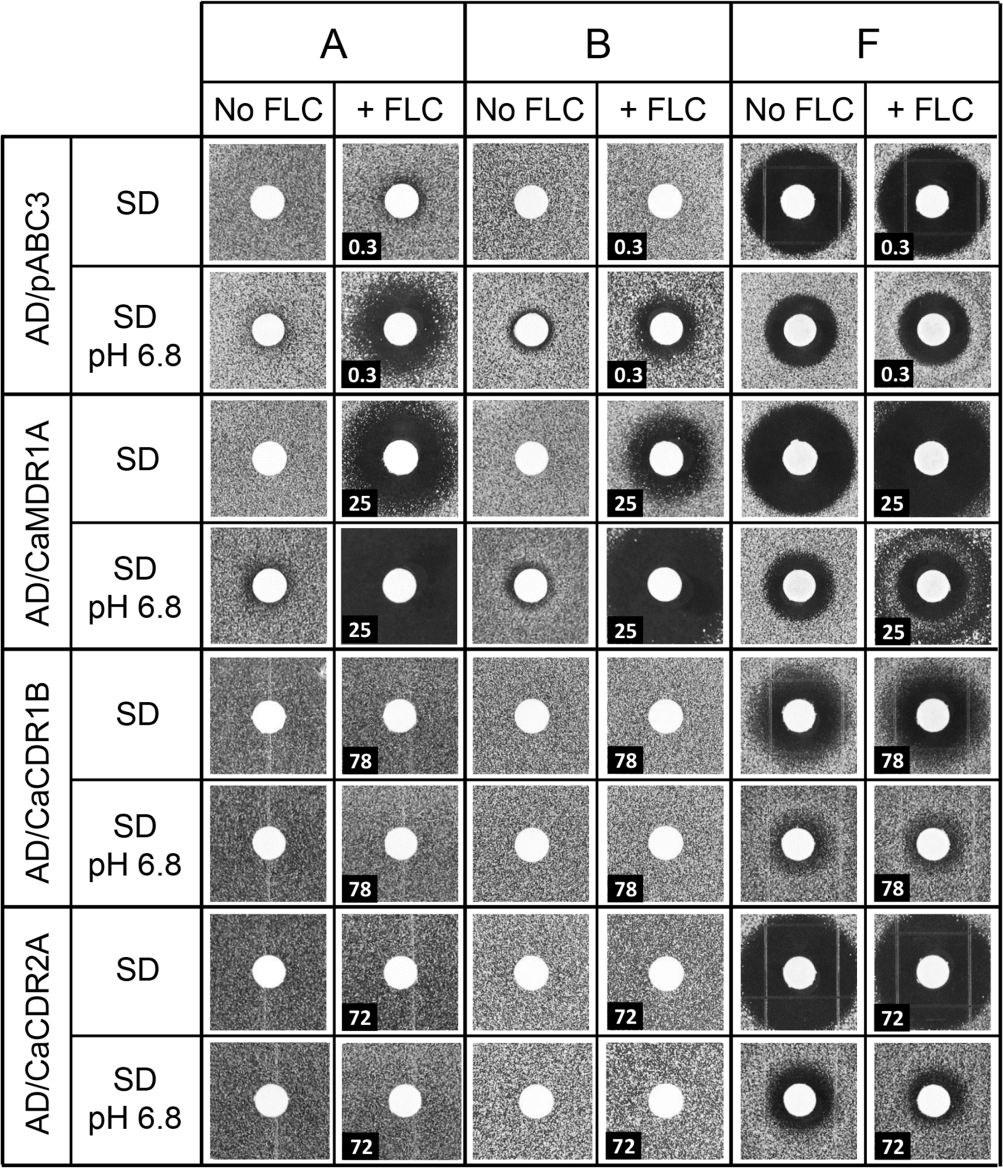
Antifungal and chemosensitization properties of compounds A, B and F tested using strains of *S*. *cerevisiae* overexpressing membrane transporters. Each compound was loaded at 50 nmol per disk. The concentration of FLC (μM) in the media (~1/8 MIC for each strain) is shown in the bottom left corner of the relevant panels.

Thirty-eight structurally-related compounds containing a cyclobutene-dione (squarile) group with substituents at positions 3 and 4 were initially screened for their ability to chemosensitize *S*. *cerevisiae* AD/CaMDR1A to FLC. The structures of the eight compounds with most relevance to the present study, with their MCC library identity (used in this paper) and IUPAC names are shown in Fig 1 (Fig 1). Their key chemical and biological properties are summarized in Table 2. Chemosensitization of yeast strains to FLC (pump inhibition) was detected by the inclusion of a suitable sub-MIC concentration of FLC in the agar used in agarose diffusion susceptibility assays. A primary screen was used to identify compounds (50 nmol applied per disk) that chemosensitized *S*. *cerevisiae* strain AD/CaMDR1 to FLC, with AD/pABC3 acting as a negative control (no heterologous pump expressed). Strain AD/CaCDR1 or AD/CaCDR2 were used in counterscreens to identify those CaMdr1p-inhibiting compounds that also affected CaCdr1p or CaCdr2p, respectively. Assays were carried out using both SD and SD pH 6.8 media. Although host environments are likely to be buffered near neutrality, unbuffered medium was also used as high microorganism concentrations because entrapment in biofilms may significantly lower the environmental pH and hence modify the plasma membrane electrochemical gradient important for Mdr1p function. The compounds which increased the sensitivity of the strain overexpressing CaMdr1p to FLC (the zones of inhibition were larger with FLC in the media) were considered as a putative inhibitors of that pump. Compounds which caused growth inhibition in the host strain (in the absence of FLC) were considered to be fungistatic or possibly fungicidal for yeast. Those growth inhibitory compounds that were rendered less effective against *S*. *cerevisiae* by the expression of an efflux pump were considered to be potential pump substrates. Only three compounds either strongly chemosensitized AD/CaMDR1 to FLC (A and B), or had antifungal activity (F) in disk diffusion assays (Fig 2) and these compounds were selected for detailed study.

**Table 2:** Summary of compound structures and properties.

MCC Compound number	Compound name	Squarile^a^ ligand 1	Squarile^a^ ligand 2	Key biological properties
**MCC1189**	A	phenylamino-	Heterocycle 2^b^	Strong CaMdr1p chemosensitization activity in SD pH 6.8 (less chemosensitization in SD). Good solubility, no antifungal activity.
**MCC1375**	B	benzylamino-	Heterocycle 2^b^	Strong CaMdr1p chemosensitization activity in SD pH 6.8 (less chemosensitization in SD). Low solubility, weak antifungal activity. Possible substrate of CaCdr1p and CaCdr2p at pH 6.8.
**MCC1484**	C	phenylamino-	Heterocycle 1^c^	Moderate CaMdr1p chemosensitization activity in SD pH 6.8 (same chemosensitization in SD).
**MCC1483**	D	benzylamino-	Heterocycle 1^c^	Moderate CaMdr1p chemosensitization activity in SD pH 6.8 only.
**MCC1194**	E	phenyl-thio-	Heterocycle 2^b^	Low chemosensitization activity, no antifungal activity, unstable.
**MCC2451**	F	chlorine	Heterocycle 2^b^	Antifungal activity in SD. Less antifungal activity in SD pH 6.8. Substrate of CaCdr1p.
**MCC1190**	G	p-Cl-phenylamino-	Heterocycle 2^b^	Inactive
**MCC1376**	H	p-methyl-phenylamino-	Heterocycle 2^b^	Inactive

^**a**^ Squarile: cyclobutene-dione

^b^ Heterocycle 2: trimethyl-ylidene-dihydroindole-

^c^ Heterocycle 1: 2-4-dimethylpyrazol-yl-

### Chemosensitization by A and B

The host strain AD/pABC3 showed background chemosensitization to FLC in the presence of compounds A and B due to the low level expression of endogenous efflux pumps, including the CaMdr1p orthologue ScFlr1p. Compounds A and B showed enhanced chemosensitization activity with strain AD/CaMDR1 (Fig 2). These chemosensitization activities were pH-dependent with greater effect at pH 6.8. Compound A gave larger inhibition zones than compound B in both unbuffered medium and at pH 6.8. Compound A at 50 nmol/disk showed no detectable antifungal activity against AD/pABC3 and, in the absence of FLC, compound B slightly inhibited growth but only in SD pH 6.8 (Fig 2 and Table 3). This growth inhibitory activity was not seen with strains overexpressing CaCdr1p or CaCdr2p, indicating that B may be a substrate of these ABC transporters. As compound A was not toxic under the conditions tested, it was not possible to evaluate whether it was a transport substrate. Serial dilution experiments in SD showed that compounds A and B were still active when as little 0.8–1.6 nmol was applied per disk. Compound B produced significantly smaller growth inhibition zones than compound A, especially above 12.5 nmol/disk, probably because B has a lower solubility in water than A.

**Table 3:** Effects of A, B and F on CaMdr1p overexpressing *S*. *cerevisiae* strain AD/CaMDR1A.

Compound	Solubility in DMSO (mM)	Antifungal activity^a^	CaMdr1p chemo-sensitization^b^(nmol/disk)	MIC_F/C_ (μM)	FIC_F_	MIC_C/F_ (μM)	FIC_C_	FICI
**A**	>20	-/-	3.2/1.6	10	0.03	0.63	<0.02^c^	<0.05^d^
**B**	~10	-/7	0.8/0.4	10	0.03	1.3	<0.03 ^c^	<0.06^d^
**F**	>20	20/14	ND	ND	ND	ND	ND	ND

^a^ Antifungal activity expressed as the diameters (mm) of inhibition zones in disk diffusion assays for SD/SD pH 6.8. “-” sign: no activity detected as the inhibition zone diameter was ≤ 6 mm (size of the disk).

^b^ Amount of compound required for detectable chemosensitization in SD/SD pH 6.8 containing 25 μM FLC (1/8^th^ AD/CaMDR1A MIC of FLC).

^c^ Growth inhibition not detected at highest concentration of compound used.

^d^ Synergistic effect

ND: not determined

### Antifungal activity of F

Compound F showed antifungal activity against all strains in the absence of FLC (Fig 2). The antifungal activity was greater in SD medium than in SD pH 6.8. The growth inhibitory zones obtained with AD/pABC3, AD/CaCDR1 and AD/CaCDR2 were unaffected by the addition of FLC. Possible chemosensitization was indicated by the slight increase in the size, or altered nature, of the zone of growth inhibition of AD/CaMDR1 caused by F when medium contained FLC (Fig 2). There was less growth inhibition of AD/CaCDR1 and AD/CaCDR2 by F, compared to inhibition of AD/pABC3 when the medium was adjusted to pH 6.8. This suggests that F had antifungal activity and was not transported effectively by CaMdr1p. It was not transported by CaCdr2p in SD, but was a weak substrate of CaCdr1p and possibly CaCdr2p at pH 6.8 (Fig 2, Table 4). The shared squarile group and/or ligand 2 (heterocycle 2) of B and F may enable their efflux by CaCdr1p and CaCdr2p.

**Table 4:** Effects of A, B and F on ABC and MFS efflux pumps overexpressed in *S*. *cerevisiae*.

Overexpressed pump	Compound	SD		SD pH 6.8	
		Substrate	Inhibitor^b^	Substrate	Inhibitor^b^
**CaMdr1p**	A	ND^a^	Yes	ND^a^	Yes
	B	ND^a^	Yes	No	Yes
	F	No	Weak	No	No
**CaCdr1p**	A	ND^a^	No	ND^a^	No
	B	ND^a^	No	Yes	No
	F	Weak	No	Weak	No
**CaCdr2p**	A	ND^a^	No	ND^a^	No
	B	ND^a^	No	Yes	No
	F	No	No	Weak	No

^a^ Not determined as the compound at 50 nmol/disk did not inhibit growth in the absence of FLC

^b^ Pump inhibitor (chemosensitizer) i.e. size of inhibition zone is increased in the presence of FLC

### Synergy of compounds with FLC

Checkerboard susceptibility assays were used to investigate any synergy between compounds and FLC. The FIC and FICI of each compound in SD buffered to pH 6.8 was measured. A and B gave FIC and FICI values that indicate strong synergy with FLC (Table 3). Compound B gave paradoxical growth at >5 μM, possibly due to limited solubility in aqueous solution [33,34]. An alternative explanation for the paradoxical growth is a poorly understood strain-specific metabolic response as has previously been reported for echinocandins [33,34]. The checkerboard assays also showed that A and B were equally effective chemosensitizers at low concentrations in liquid media. These results suggest that the chemosensitization shown by B in solidified medium may have been adversely affected by its modest solubility. The combination of FLC and compound A was fungicidal. After 48 h exposure to 1.3 μM compound A plus ~1/2 MIC of FLC <1% of AD/CaMDR1 cells were viable on non-selective medium.

### Susceptibility of *C*. *albicans* strains to compound A

**Fig 3:**
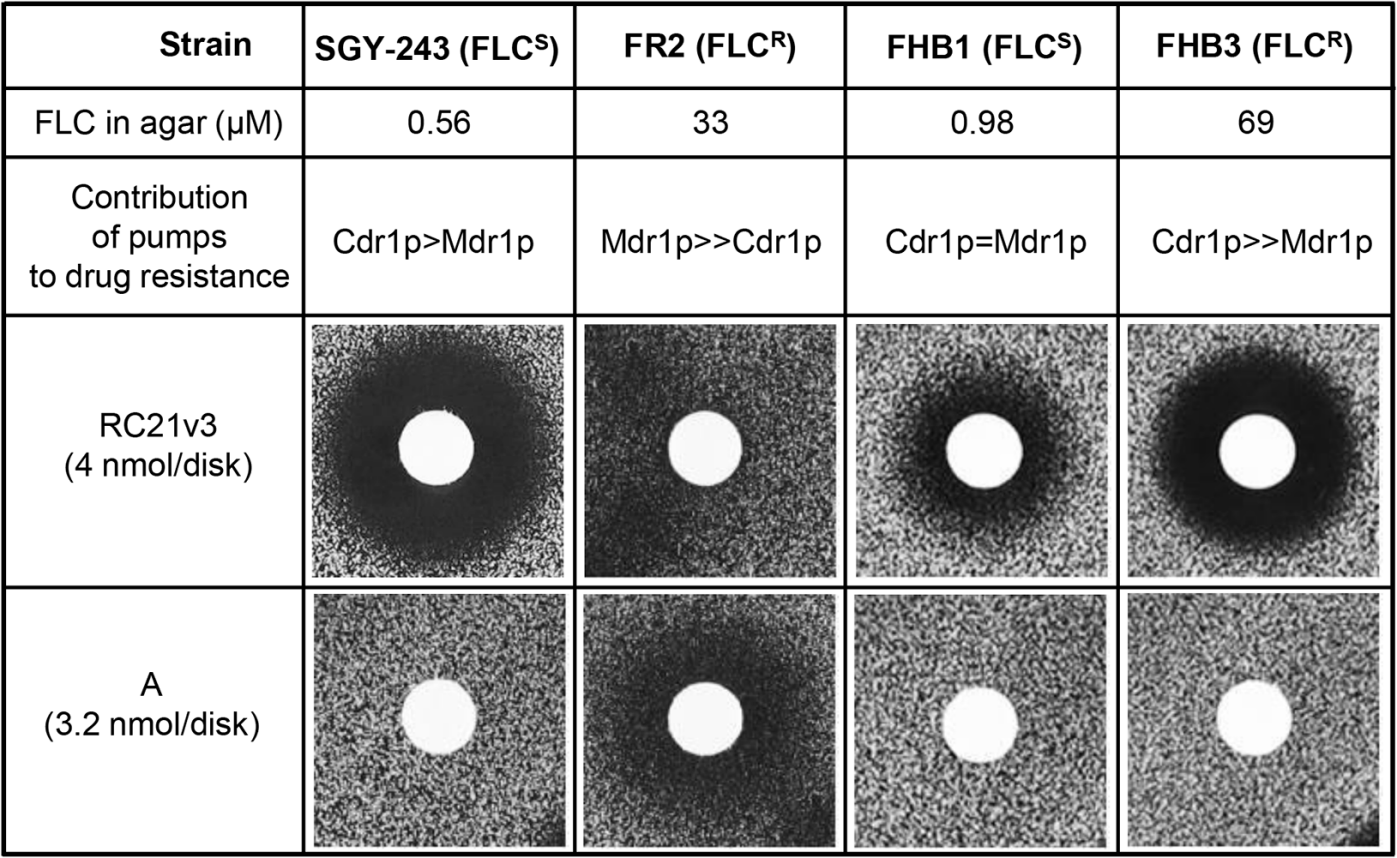
Chemosensitization of *C*. *albicans* to FLC. The strains used and conditions for the agarose diffusion assays are described in Materials and methods. FLC^S^—susceptible to FLC; FLC^R^—resistant to FLC.

The effect of compound A on the fungal pathogen *C*. *albicans* was tested using two pairs of genetically related strains. For each pair one was FLC-susceptible and the other a FLC-resistant daughter strain (Fig 3). The azole resistance of FR2 is primarily CaMdr1p dependent [29] whereas the azole resistance of FHB3 is CaCdr1p/CaCdr2p dependent [35]. The peptide derivative RC21v3 [18] was used as a positive control for CaCdr1p-specific chemosensitization. Compound A showed no detectable antifungal activity in the absence of FLC. Both parental *C*. *albicans* strains, SGY-243 and FHB1, were chemosensitized to FLC by the CaCdr1p inhibitor RC21v3 but not by A due to the low-level constitutive expression of Cdr1p in SGY-243 and FHB1 [29]. Similarly, the Cdr1p-dependent FLC resistance of FHB3 was chemosensitized by RC21v3 but not by A. In contrast, the Mdr1p-dependent FLC resistance of the FR2 strain was chemosensitized by A but not by RC21v3. These results show that compound A chemosensitizes *C*. *albicans* clinical isolates overexpressing Mdr1p, but not Cdr1p, to FLC.

### Compound A inhibits pumping activity of CaMdr1p

**Fig 4:**
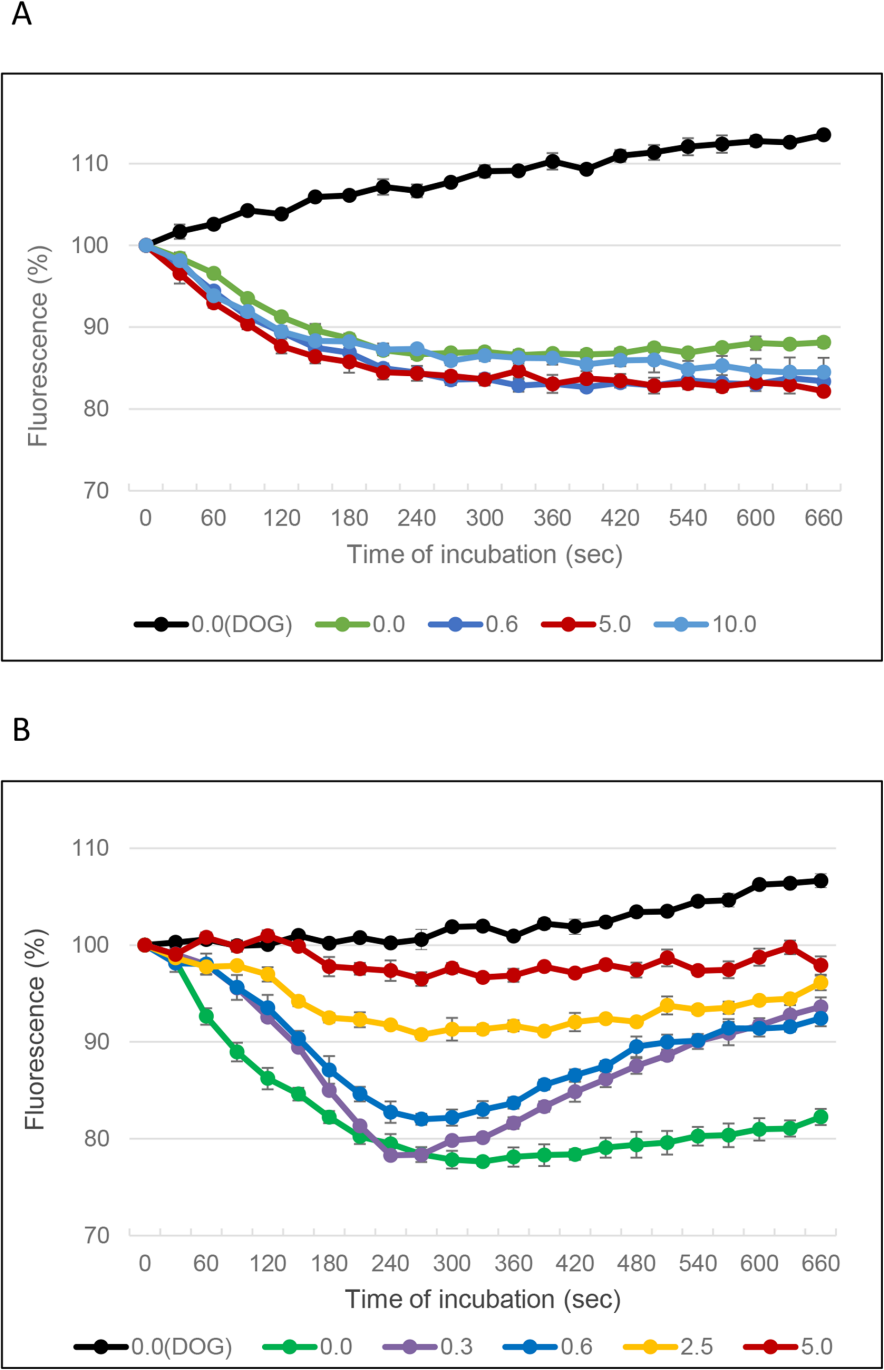
Effect of compound A on Nile Red efflux from *S*. *cerevisiae* cells expressing CaCdr1p or CaMdr1p membrane transporters. A: AD/CaCDR1; B: AD/CaMDR1. Efflux of Nile Red was initiated by adding glucose (50 μl 80 mM) at time 0 (except for black line which represents cells to which DOG was added rather than glucose, and no compound A was added). Cell-associated Nile Red fluorescence was measured. Legend: concentration of compound A in μM. Data are representative of 3 separate experiments.

The effect of compound A on the glucose-dependent efflux of Nile Red by either CaMdr1p or CaCdr1p overexpressed in *S*. *cerevisiae* was determined [25]. For both transporters, maximal Nile Red export was achieved 4–5 minutes after cell energisation at 26°C, and the cells maintained this equilibrium for at least a further 5 min (Fig 4). In contrast, the control strain AD/pABC3 did not show energy-dependent Nile Red efflux in either the absence or presence of compound A. These results indicate that endogenous ScFlr1p activity was not detected.

Nile Red efflux by CaCdr1p was not inhibited by compound A at 10 μM (Fig 4A). In contrast, energy-dependent pumping of Nile Red by CaMdr1p was completely inhibited (fluorescence >90% of the initial value) at ≥2.5 μM compound A (Fig 4B). At 0.3 μM compound A, Nile Red pumping was initially unaffected, but by 10 min the fluorescence of the dye returned to near starting values, indicative of dye uptake. The initial kinetics of Nile Red efflux showed dose-dependent inhibition at compound A concentrations between 0.3 and 2.5 μM. This response was unaffected when the preincubation with the inhibitor was extended from 3 to 6 min. These results suggest that the inactivation of the CaMdr1p transporter by compound A requires the active turnover of the pump.

### Inhibition of drug efflux by compound A is independent of plasma membrane potential

**Fig 5:**
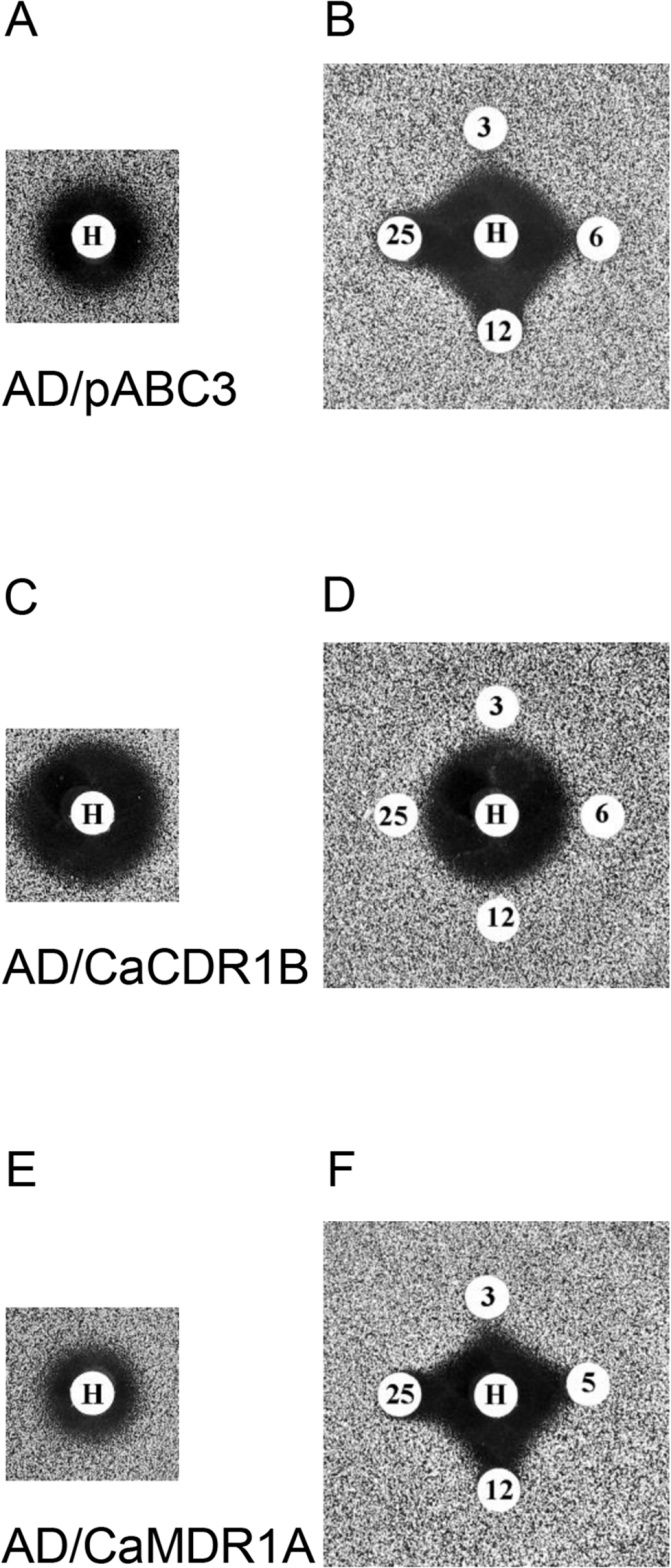
Effect of compound A on hygromycin B toxicity in agarose diffusion susceptibility assays. A, B: host strain AD/pABC3; C, D: AD/CaCDR1; and E, F: AD/CaMDR1 at pH 6.8. A, C, E: paper disks loaded with hygromycin B (H; 200 nmol/disk); B, D, F: paper disks loaded with hygromycin B (H; 200 nmol/disk) or compound A: 3, 6, 12 or 25 nmol/disk.

The uptake and toxicity of the cationic inhibitor of protein synthesis hygromycin B depends on the electrochemical gradient of the plasma membrane [36]. Hygromycin B has greater toxicity for cells over-expressing the ABC transporter CaCdr1p than the host strain (compare the zone of growth inhibition on Fig 5A with that on Fig 5C). This is due to the electrogenic nature of CaCdr1p; the ATP hydrolysis it catalyses generates protons and the substrates it transports are partially positively charged [37]. The growth inhibitory zone for the AD/CaCDR1 strain exposed to 200 nmol hygromycin B was significantly larger than for AD/pABC3 and its size was not modified by placing adjacent disks containing up to 25 nmol compound A (Fig 5D). This result shows that compound A does not inhibit CaCdr1p or directly modify the plasma membrane electrochemical gradient. In contrast, CaMdr1p utilises membrane potential and, as expected, its overexpression caused reduced susceptibility to hygromycin B (compare the zone of growth inhibition on Fig 5E with that on Fig 5A). Furthermore, the initially circular zone of hygromycin B-dependent growth inhibition zone was extended, in a dose-dependent manner, towards disks containing as little 3 nmol of compound A (Fig 5F), indicating synergism. This result showed that inactivation of CaMdr1p by compound A, as expected, either increased the electrochemical gradient or reduced CaMdr1p-dependent efflux of hygromycin B. We also note that the control strain AD/pABC3 (Fig 5B) showed the same pattern of hygromycin sensitivity as AD/CaMDR1 (Fig 5F). This result is consistent with compound A inhibiting the endogenous Flr1p expressed by AD/pABC3 in a dose-dependent manner.

### Toxicity to cultured human cells

Compounds A and F at 100 μM displayed minor toxicity towards HEp2 cells after 4 h (0.3% dead for both compounds, 0.1% and 0.3% damaged cells for A and F, respectively). After 24 h ~94% of HEp2 cells treated with compound A and 99% of HEp2 cells treated with compound F were viable.

## Discussion

Our combinatorial library, based on a derivatized squarile (cyclobutene-dione) core, contained compounds with a variety of antifungal and chemosensitization properties. Compounds A and B, the best chemosensitizers of strain AD/CaMDR1, contain heterocyclic substituents at atoms 3 and 4 of the squarile core (Fig 1, Table 2). Both compounds were more active in SD buffered to pH 6.8 than in unbuffered SD (initially pH ~6.0). Compound A appeared to be a true chemosensitizer and was without antifungal activity against *S*. *cerevisiae*. It was the most potent inhibitor of CaMdr1p and its FICI at pH 6.8 of <0.05 indicates strong synergy with FLC (Table 3).

Compound A also showed no antifungal activity with clinical isolates of *C*. *albicans* (Fig 3). Although compound B gave comparable chemosensitization of AD/CaMDR1 to FLC as A, it showed slight antifungal activity at higher pH and was a substrate of CaCdr1p and CaCdr2p. The lower solubility of B resulted in paradoxical growth in checkerboard assays at concentrations >5 μM in the presence of FLC. These properties indicate that compound A is more promising than B for further development as a chemosensitizer, despite being unable to exclude A as a substrate of the efflux pumps CaCdr1p and CaCdr2p.

C and D were the smallest compounds in our library (Fig 1) that chemosensitized AD/CaMDR1 to FLC (Table 2). Atom 3 of the squarile core bears phenylamine as ligand 1 in C and benzylamine in D, with C showing the greater chemosensitization of AD/CaMDR1 to FLC. The presence of a larger heterocycle on atom 4 on the squarile group in A and B conferred significantly greater chemosensitization than the 2-4-dimethylpyrazol-yl heterocycle on C and D. Substitution of the benzene para-position hydrogen with a methyl group (H) or chlorine atom (G) rendered A inactive. Replacement of the linking nitrogen in A with sulphur in E conferred low activity, probably due to instability as the phenyl-thiol derivative gradually changed color from light pink to red. The chlorine-containing compound F possesses the smallest derivative of the ligand 1 (Fig 1, Table 2). This compound is not toxic for cultured human cells. It showed antifungal activity, but lack of synergy with FLC (Fig 2) indicated that it is unlikely to affect the same target as FLC or inhibit pump-mediated efflux. F was not a substrate of CaMdr1p or CaCdr2p (at lower pH) but appeared to be a weak substrate of CaCdr1p (Fig 2, Table 4). The specificity of compound F as a substrate of CaCdr1p indicates that modification of the squarile group and/or the ligand 2 heterocyle 2 may help future chemosensitizer design.

The first in class small molecule A appears to be a relatively specific inhibitor of drug efflux by interacting with the MFS efflux pump CaMdr1p. It inhibits Nile Red efflux by CaMdr1p but not by CaCdr1p, and does so without directly affecting plasma membrane electrochemical potential. Although this low molecular weight MFS inhibitor is synergistic with FLC, and this combination was fungicidal, compound A cannot yet be excluded as a substrate of the ABC transporters CaCdr1p and CaCdr2p. We envisage the use of compound A as a conformation stabilizer in structural studies of CaMdr1p and to assay, in combination with the CaCdr1p-specific inhibitor RC21v3 [18,38], the relative contributions of CaCdr1p- and CaMdr1p-mediated drug efflux in clinical isolates [24]. A related next-generation chemosensitizer that is demonstrably not a fungal ABC transporter substrate will be needed before this class of compound can be applied in the clinic. Such compounds could be used to sensitize to FLC the subset of strains overexpressing CaMdr1p. By inhibiting endogenous CaMdr1p activity, compound A might also be used to test the relationship between CaMdr1p function and the pathogenicity of *C*. *albicans* strains in general [10,11].

## References

[1] Akins RA (2005) An update on antifungal targets and mechanisms of resistance in *Candida albicans* . Med Mycol 43: 285–318. 16110776 10.1080/13693780500138971

[2] Cannon RD, Lamping E, Holmes AR, Niimi K, Baret PV, Keniya MV, et al (2009) Efflux-mediated antifungal drug resistance. Clin Microbiol Rev 22: 291–321. 10.1128/CMR.00051-08 19366916 PMC2668233

[3] Holmes AR, Lin YH, Niimi K, Lamping E, Keniya M, Niimi M, et al (2008) ABC transporter Cdr1p contributes more than Cdr2p does to fluconazole efflux in fluconazole-resistant *Candida albicans* clinical isolates. Antimicrob Agents Chemother 52: 3851–3862. 10.1128/AAC.00463-08 18710914 PMC2573144

[4] Perea S, Lopez-Ribot JL, Kirkpatrick WR, McAtee RK, Santillan RA, Martinez M, et al (2001) Prevalence of molecular mechanisms of resistance to azole antifungal agents in *Candida albicans* strains displaying high-level fluconazole resistance isolated from human immunodeficiency virus-infected patients. Antimicrob Agents Chemother 45: 2676–2684. 11557454 10.1128/AAC.45.10.2676-2684.2001 PMC90716

[5] Lopez-Ribot JL, McAtee RK, Lee LN, Kirkpatrick WR, White TC, Sanglard D, et al (1998) Distinct patterns of gene expression associated with development of fluconazole resistance in serial *Candida albicans* isolates from human immunodeficiency virus-infected patients with oropharyngeal candidiasis. Antimicrob Agents Chemother 42: 2932–2937. 9797228 10.1128/aac.42.11.2932 PMC105968

[6] White TC (1997) Increased mRNA levels of ERG16, CDR, and MDR1 correlate with increases in azole resistance in *Candida albicans* isolates from a patient infected with human immunodeficiency virus. Antimicrob Agents Chemother 41: 1482–1487. 9210670 10.1128/aac.41.7.1482 PMC163944

[7] White TC, Holleman S, Dy F, Mirels LF, Stevens DA (2002) Resistance mechanisms in clinical isolates of *Candida albicans* . Antimicrob Agents Chemother 46: 1704–1713. 12019079 10.1128/AAC.46.6.1704-1713.2002 PMC127245

[8] Franz R, Kelly SL, Lamb DC, Kelly DE, Ruhnke M, Morschhauser J (1998) Multiple molecular mechanisms contribute to a stepwise development of fluconazole resistance in clinical *Candida albicans* strains. Antimicrob Agents Chemother 42: 3065–3072. 9835492 10.1128/aac.42.12.3065 PMC106000

[9] Fling ME, Kopf J, Tamarkin A, Gorman JA, Smith HA, Koltin Y (1991) Analysis of a *Candida albicans* gene that encodes a novel mechanism for resistance to benomyl and methotrexate. Mol Gen Genet 227: 318–329. 2062311 10.1007/BF00259685

[10] Becker JM, Henry LK, Jiang W, Koltin Y (1995) Reduced virulence of *Candida albicans* mutants affected in multidrug resistance. Infection and immunity 63: 4515–4518. 7591094 10.1128/iai.63.11.4515-4518.1995 PMC173643

[11] Lohberger A, Coste AT, Sanglard D (2014) Distinct roles of *Candida albicans* drug resistance transcription factors TAC1, MRR1, and UPC2 in virulence. Eukaryot Cell 13: 127–142. 10.1128/EC.00245-13 24243794 PMC3910953

[12] Dunkel N, Blass J, Rogers PD, Morschhauser J (2008) Mutations in the multi-drug resistance regulator MRR1, followed by loss of heterozygosity, are the main cause of MDR1 overexpression in fluconazole-resistant *Candida albicans* strains. Mol Microbiol 69: 827–840. 10.1111/j.1365-2958.2008.06309.x 18577180 PMC2678921

[13] Morschhauser J (2010) Regulation of multidrug resistance in pathogenic fungi. Fungal Genet Biol 47: 94–106. 10.1016/j.fgb.2009.08.002 19665571

[14] Hiller D, Sanglard D, Morschhauser J (2006) Overexpression of the MDR1 gene is sufficient to confer increased resistance to toxic compounds in *Candida albicans* . Antimicrob Agents Chemother 50: 1365–1371. 16569853 10.1128/AAC.50.4.1365-1371.2006 PMC1426927

[15] Cheng S, Clancy CJ, Nguyen KT, Clapp W, Nguyen MH (2007) A *Candida albicans* petite mutant strain with uncoupled oxidative phosphorylation overexpresses MDR1 and has diminished susceptibility to fluconazole and voriconazole. Antimicrob Agents Chemother 51: 1855–1858. 17325226 10.1128/AAC.00182-07 PMC1855545

[16] Niimi M, Niimi K, Takano Y, Holmes AR, Fischer FJ, Uehara Y, et al (2004) Regulated overexpression of CDR1 in *Candida albicans* confers multidrug resistance. Journal of Antimicrobial Chemotherapy 54: 999–1006. 15486081 10.1093/jac/dkh456

[17] Monk BC, Niimi K, Lin S, Knight A, Kardos TB, Cannon RD, et al (2005) Surface-active fungicidal D-peptide inhibitors of the plasma membrane proton pump that block azole resistance. Antimicrobial Agents & Chemotherapy 49: 57–70. 15616276 10.1128/AAC.49.1.57-70.2005 PMC538910

[18] Niimi K, Harding DR, Holmes AR, Lamping E, Niimi M, Tyndall JD, et al (2012) Specific interactions between the *Candida albicans* ABC transporter Cdr1p ectodomain and a D-octapeptide derivative inhibitor. Mol Microbiol 85: 747–767. 10.1111/j.1365-2958.2012.08140.x 22788839 PMC3418399

[19] Schuetzer-Muehlbauer M, Willinger B, Egner R, Ecker G, Kuchler K (2003) Reversal of antifungal resistance mediated by ABC efflux pumps from *Candida albicans* functionally expressed in yeast. Int J Antimicrob Agents 22: 291–300. 13678837 10.1016/s0924-8579(03)00213-9

[20] Sharma M, Manoharlal R, Shukla S, Puri N, Prasad T, Ambudkar SV, et al (2009) Curcumin modulates efflux mediated by yeast ABC multidrug transporters and is synergistic with antifungals. Antimicrob Agents Chemother 53: 3256–3265. 10.1128/AAC.01497-08 19470507 PMC2715616

[21] Ricardo E, Costa-de-Oliveira S, Dias AS, Guerra J, Rodrigues AG, Pina-Vaz C (2009) Ibuprofen reverts antifungal resistance on *Candida albicans* showing overexpression of CDR genes. FEMS Yeast Res 9: 618–625. 10.1111/j.1567-1364.2009.00504.x 19416368

[22] Maurya IK, Thota CK, Verma SD, Sharma J, Rawal MK, Ravikumar B, et al (2013) Rationally designed transmembrane peptide mimics of the multidrug transporter protein Cdr1 act as antagonists to selectively block drug efflux and chemosensitize azole-resistant clinical isolates of *Candida albicans* . The Journal of biological chemistry 288: 16775–16787. 10.1074/jbc.M113.467159 23592791 PMC3675610

[23] Diwischek F, Morschhauser J, Holzgrabe U (2009) Cerulenin analogues as inhibitors of efflux pumps in drug-resistant *Candida albicans* . Arch Pharm (Weinheim) 342: 150–164. 10.1002/ardp.200800160 19253321

[24] Holmes AR, Keniya MV, Ivnitski-Steele I, Monk BC, Lamping E, Sklar LA, et al (2012) The monoamine oxidase A inhibitor clorgyline is a broad-spectrum inhibitor of fungal ABC and MFS transporter efflux pump activities which reverses the azole resistance of *Candida albicans* and *Candida glabrata* clinical isolates. Antimicrob Agents Chemother 56: 1508–1515. 10.1128/AAC.05706-11 22203607 PMC3294898

[25] Ivnitski-Steele I, Holmes AR, Lamping E, Monk BC, Cannon RD, Sklar LA (2009) Identification of Nile red as a fluorescent substrate of the *Candida albicans* ATP-binding cassette transporters Cdr1p and Cdr2p and the major facilitator superfamily transporter Mdr1p. Analytical biochemistry 394: 87–91. 10.1016/j.ab.2009.07.001 19577533 PMC2739806

[26] Lamping E, Monk BC, Niimi K, Holmes AR, Tsao S, Tanabe K, et al (2007) Characterization of three classes of membrane proteins involved in fungal azole resistance by functional hyperexpression in *Saccharomyces cerevisiae* . Eukaryot Cell 6: 1150–1165. 17513564 10.1128/EC.00091-07 PMC1951111

[27] Marr KA, Lyons CN, Ha K, Rustad TR, White TC (2001) Inducible azole resistance associated with a heterogeneous phenotype in *Candida albicans* . Antimicrob Agents Chemother 45: 52–59. 11120944 10.1128/AAC.45.1.52-59.2001 PMC90239

[28] Kelly R, Miller SM, Kurtz MB, Kirsch DR (1987) Directed mutagenesis in *Candida albicans*: one-step gene disruption to isolate ura3 mutants. Mol Cell Biol 7: 199–208. 3031459 10.1128/mcb.7.1.199-208.1987 PMC365057

[29] Albertson GD, Niimi M, Cannon RD, Jenkinson HF (1996) Multiple efflux mechanisms are involved in *Candida albicans* fluconazole resistance. Antimicrob Agents Chemother 40: 2835–2841. 9124851 10.1128/aac.40.12.2835 PMC163632

[30] Marchetti O, Moreillon P, Glauser MP, Bille J, Sanglard D (2000) Potent synergism of the combination of fluconazole and cyclosporine in *Candida albicans* . Antimicrobial Agents & Chemotherapy 44: 2373–2381. 10952582 10.1128/aac.44.9.2373-2381.2000 PMC90072

[31] Odds FC (2003) Synergy, antagonism, and what the chequerboard puts between them. J Antimicrob Chemother 52: 1 12805255 10.1093/jac/dkg301

[32] Niimi K, Harding DR, Parshot R, King A, Lun DJ, Decottignies A, et al (2004) Chemosensitization of fluconazole resistance in *Saccharomyces cerevisiae* and pathogenic fungi by a D-octapeptide derivative. Antimicrob Agents Chemother 48: 1256–1271. 15047528 10.1128/AAC.48.4.1256-1271.2004 PMC375246

[33] Stevens DA, Espiritu M, Parmar R (2004) Paradoxical effect of caspofungin: reduced activity against *Candida albicans* at high drug concentrations. Antimicrob Agents Chemother 48: 3407–3411. 15328104 10.1128/AAC.48.9.3407-3411.2004 PMC514730

[34] Chamilos G, Lewis RE, Albert N, Kontoyiannis DP (2007) Paradoxical effect of Echinocandins across *Candida* species in vitro: evidence for echinocandin-specific and candida species-related differences. Antimicrob Agents Chemother 51: 2257–2259. 17438060 10.1128/AAC.00095-07 PMC1891358

[35] Richards TS, Oliver BG, White TC (2008) Micafungin activity against *Candida albicans* with diverse azole resistance phenotypes. J Antimicrob Chemother 62: 349–355. 10.1093/jac/dkn156 18436555 PMC2532560

[36] McCusker JH, Perlin DS, Haber JE (1987) Pleiotropic plasma membrane ATPase mutations of *Saccharomyces cerevisiae* . Mol Cell Biol 7: 4082–4088. 2963211 10.1128/mcb.7.11.4082-4088.1987 PMC368079

[37] Milewski S, Mignini F, Prasad R, Borowski E (2001) Unusual susceptibility of a multidrug-resistant yeast strain to peptidic antifungals. Antimicrob Agents Chemother 45: 223–228. 11120970 10.1128/AAC.45.1.223-228.2001 PMC90265

[38] Hayama K, Ishibashi H, Ishijima SA, Niimi K, Tansho S, Ono Y, et al (2012) A D-octapeptide drug efflux pump inhibitor acts synergistically with azoles in a murine oral candidiasis infection model. FEMS microbiology letters 328: 130–137. 10.1111/j.1574-6968.2011.02490.x 22211961

